# The COVID‐19 Pandemic Experience of Families With Children With Developmental Disorders in Northeastern Germany

**DOI:** 10.1111/cch.70370

**Published:** 2026-11-03

**Authors:** Teresa Vela Martín, Martina P. Neininger, Gudrun E. Krause, Thilo Bertsche, Astrid Bertsche, Sarah Jeschke

**Affiliations:** ^1^ Department of Neuropediatrics, Center for Children and Adolescents University Medicine Greifswald Greifswald Germany; ^2^ Department of Neuropediatrics and Social Pediatric Center, Hospital for Children and Adolescents University Medicine Rostock Rostock Germany; ^3^ German Center for Child and Adolescent Health (DZKJ), Partner Site Greifswald/Rostock Greifswald Germany; ^4^ Department of Psychiatry and Psychotherapy Leipzig University Hospital Leipzig Germany; ^5^ Drug Safety Center, Leipzig University and Leipzig University Hospital, and Clinical Pharmacy, Institute of Pharmacy, Medical Faculty, Leipzig University Leipzig Germany

**Keywords:** COVID‐19 pandemic, developmental disorders, effects of the pandemic, Northeastern Germany, parent‐child interaction, vaccination

## Abstract

**Background:**

Developmental disorders are associated with considerable challenges for affected children and their families. During the COVID‐19 pandemic, families faced additional stress, including challenging decisions regarding COVID‐19 vaccination for their children. This exploratory study therefore examined the experiences of families with children with developmental disorders during the pandemic and their attitudes towards vaccination, including the perceived benefits and disadvantages of their decision.

**Methods:**

This exploratory study was conducted from April to June 2022 at a university hospital in Northeastern Germany. The semi‐structured interviews included open and closed questions, as well as 5‐point Likert scales.

**Results:**

We interviewed 65 parents of children aged 3–17 years (median age: 6; Q25/Q75: 4/11). Among them, 69% reported a negative impact on their child, most frequently emotional and behavioural changes (45%), followed by developmental and educational setbacks (26%) and social and communication difficulties (14%). Seventy‐three percent of parents perceived the influence of the COVID‐19 pandemic on the parent–child interaction as very, rather or partly burdensome. Governmental support was judged as ‘not adequate’ or ‘rather not adequate’ by 64% of parents. Among the 44 children eligible for COVID‐19 vaccination, 59% were not vaccinated. Fear of adverse events was the main reason against vaccination (81%), whereas fear of COVID‐19 infection was the main motivation for those who chose to vaccinate (89%). Vaccination was associated with perceived benefits in daily life, whereas nonvaccination was associated with reduced participation and increased infection‐related worries.

**Conclusions:**

Most parents of children with developmental disorders perceived negative effects of the pandemic on the lives of their child and themselves. Among children eligible for COVID‐19 vaccination, more than half of the parents decided against vaccination, despite reported benefits of vaccination and reported disadvantages associated with nonvaccination. Future research is needed to develop tailored vaccination campaigns and safety‐focused communication strategies for children with developmental disorders.

## Summary

- COVID‐19 disruptions led to developmental stagnation and behavioural challenges in most children with developmental disorders, according to their parents.
- Many parents of children with developmental disorders found pandemic‐related governmental support not adequate, and over 70% reported increased strain in parent–child interactions during the pandemic.
- Fear of side effects outweighed perceived benefits in vaccine decisions for many families of children with developmental disorders.
- These findings highlight the need for targeted support for parents and children with developmental disorders during health crises.
- Future research is needed to develop tailored vaccination campaigns and safety‐focused communication strategies.

## 1 Introduction

Developmental disorders are common health conditions in childhood and adolescence (Li et al. 2023) and place a considerable burden on the children affected and their families (Gardiner et al. 2018). In this study, the term developmental disorders refers to mixed developmental disorders, learning disability and intellectual disability. Children with developmental disorders often have additional comorbidities, such as epilepsy, sleep problems or emotional and behavioural difficulties, which may further increase care needs and family burden (Schieve et al. 2006). As a result, these families already faced a variety of challenges in their everyday lives even before the COVID‐19 pandemic, including high caregiving demands, financial strain and increased emotional and psychosocial stress (Gardiner et al. 2018; Schieve et al. 2006; Houtrow et al. 2025; Irazábal et al. 2012).

In Northeastern Germany, families experienced fast‐changing contact and quarantine rules, mask requirements and restrictions in school and early childhood education between 2020 and 2022 (Grote et al. 2021). Depending on local infection rates, school attendance could temporarily shift between regular, restricted and online formats (Heinsohn et al. 2022; Sombetzki et al. 2021). The negative effects of these measures on the mental health of children and their families have been widely reported in general populations worldwide (Onyeaka et al. 2021; Miao et al. 2023). Studies of children with developmental disorders and their families suggest that some children experienced negative effects similar to typically developing children (Miao et al. 2023; Bussières et al. 2021), whereas other children remained stable or even benefited from the pandemic (Bussières et al. 2021; Yusuf et al. 2022). In addition to psychosocial challenges, COVID‐19 is associated with considerable health risks for individuals with developmental disorders, particularly those with intellectual disabilities (Courtenay and Perera 2020). Compared to the general population, they face an increased risk of severe COVID‐19 outcomes, including hospitalisation and mortality (Courtenay and Perera 2020; Landes et al. 2020; Turk et al. 2020).

Moreover, the COVID‐19 vaccination of children became an important and sensitive issue for parents (Kafadar et al. 2023; Kraaijeveld et al. 2022). In Germany, the Standing Committee on Vaccination recommended vaccination for children aged 5 years and older with pre‐existing conditions in December 2021 (Vygen‐Bonnet et al. 2022). In May 2022, the recommendation was expanded to include healthy children aged 5 years and older (Vygen‐Bonnet et al. 2022). Studies in the general population showed that parental vaccine acceptance and decision‐making were influenced by sociodemographic, psychological and contextual factors, including socio‐economic status, cultural background and individual risk perceptions (van de Berg et al. 2024; Purrmann et al. 2021; Brandstetter et al. 2022; Galanis et al. 2022; Blackwell et al. 2025; Lazarus et al. 2024). Among parents of children with developmental disorders, concerns such as potential side effects and the novelty of the vaccine have been reported (Bonuck et al. 2022).

However, data on pandemic‐related experiences, vaccination decisions and related consequences and parental evaluation of governmental support among families of children with developmental disorders are scarce. This gap in research is particularly relevant as these children are at increased risk of severe illness from COVID‐19 (Courtenay and Perera 2020; Landes et al. 2020; Turk et al. 2020; Tinker et al. 2021) and are especially vulnerable to changes in their daily routines (Aishworiya and Kang 2021; Summers et al. 2021). This study explored additional constraints of the COVID‐19 pandemic on the already difficult everyday lives of parents of children with developmental disorders. Although several years have passed since the acute phase of the COVID‐19 pandemic, its long‐term consequences for these children and their families persist and are still being studied (Arbeau et al. 2025). Data from the active pandemic phase therefore remain important for understanding how families of children with developmental disorders navigated vaccination decisions and service disruptions, and for developing measures to cope with future public health crises.

Against this background, this study aimed to explore the experiences of families with children with developmental disorders during the COVID‐19 pandemic in a region in Northeastern Germany, focusing on perceived impact and burden, parents' assessment of governmental support, attitudes towards COVID‐19 vaccination and the perceived benefits and disadvantages of their decision.

## 2 Methods

### 2.1 Patients and Setting

After approval of the exploratory study by the responsible ethics committee (Registration Number: A 2022‐0067, approval granted on 01.04.2022), semi‐structured interviews were performed with parents of children with developmental disorders from April 2022 to June 2022. All parents of children aged 3–18 years with a current diagnosis of a mixed specific developmental disorder (F83), a learning disability (F81.9) or intellectual disability (F70, F71, F72 or F73) treated at the social paediatric centre (multidisciplinary paediatric neurology and developmental department) of a university hospital in Northeastern Germany were invited. In this context, mixed developmental disorder refers to clinically relevant delays in more than one developmental domain (language, cognition or motor skills) and applies to children aged younger than 6 years. Among children aged older than 6 years, learning disability applies to children with learning difficulties and an IQ between 70 and 85, whereas intellectual disability refers to an IQ below 70. For better readability, throughout the manuscript those diagnoses are summarised under the term developmental disorders. Eligible parents were recruited consecutively at the social paediatric centre during their child's outpatient care and were directly asked in person whether they would like to participate. Only one parent was interviewed per family. Each participant was interviewed by the same medical student in the physician's office or a nearby room to ensure equal conditions for all participants. Participants had to be the legal guardians with sufficient German language skills, and their children had to attend school or early education. Participation was voluntary and informed consent was obtained from all parents.

### 2.2 Data Assessment

Since no validated questionnaire was available for the research question and due to the exploratory nature of the study, a questionnaire for the semi‐structured interviews was designed by the study team. The study team consisted of a neuropaediatrician, a child and adolescent psychotherapist, a pharmacist having expertise with paediatric patients and a medical student. The questionnaire was piloted with two parents of healthy children. Based on this pilot, the clarity, comprehensibility and practicability of the interview were improved. The questionnaire ensured that every parent was presented with the same questions in the same order. It included both open and closed questions, as well as 5‐point Likert scales. The survey had two sections: The first section, focusing on the perceived impact of the COVID‐19 pandemic on the family's and child's daily life, was completed by all parents. The second section focused on COVID‐19 vaccination and was only answered by parents whose children were eligible for vaccination. Within this section, questions differed depending on whether the child had been vaccinated or not.

1. Perceived impact of the COVID‐19 pandemic on the family's and child's daily life
  1.1 In your opinion, did the COVID‐19 pandemic have an impact on your child? If so, what kind of impact? (multiple answers possible)
  1.2 How burdensome was the COVID‐19 pandemic with regard to the parent–child interaction? (Likert scale ranging from *not at all*, *rather not*, *partly burdensome/partly not burdensome*, *rather*, to *very*)
  1.3 How adequate would you rate the governmental support for families with a child with developmental disorders during the COVID‐19 pandemic? (Likert scale ranging from *not at all*, *rather not*, *partly adequate/partly inadequate*, *rather*, to *very*)
2. Perspectives on the vaccination against COVID‐19
  2.1 Is your child vaccinated against COVID‐19? (closed question, yes/no)
    - [if vaccinated] What were your reasons for vaccination? The participants were presented with a list of 4 reasons: Fear of consequences of a COVID‐19 infection, vaccination considered necessary, solidarity towards the population and explicit medical recommendation. Besides, the parents had the opportunity to add further reasons. (multiple answers possible)
    - [if not vaccinated] What were your reasons against vaccination? The participants were presented with a list of 4 reasons: Fear of adverse events, uncertainty about effectiveness, vaccination considered unnecessary and explicit medical contraindication. Besides, the parents had the opportunity to add further reasons. (multiple answers possible)
  2.2Perceived benefits and disadvantages of the vaccination
    - [if vaccinated] Did the decision for the vaccination have benefits for your child? If so, what were they? (multiple answers possible)
    - [if vaccinated] Did the decision for the vaccination have disadvantages for your child? If so, what were they? (multiple answers possible)
    - [if not vaccinated] Did the decision against the vaccination have benefits for your child? If so, what were they? (multiple answers possible)
    - [if not vaccinated] Did the decision against the vaccination have disadvantages for your child? If so, what were they? (multiple answers possible)

Patients' characteristics were collected from their medical files. We also asked parents about their child's period of absence from the educational institution due to closure during the pandemic. Further, we collected sociodemographic data from the parents. Sociodemographic data included household composition, whether more than one child lived in the household, and employment status.

### 2.3 Data Analysis

Quantitative data were analysed descriptively using Microsoft Excel, version 16.90.2. The responses to the open questions were documented and subsequently clustered by the study team. The categories were derived from the parents' answers. Each response was independently assigned to the derived categories by all members of the expert panel. In cases of divergent category assignments, discrepancies were resolved through discussion among the panel members until consensus was reached. Therefore, inter‐rater agreement was not calculated.

## 3 Results

### 3.1 Participants' Characteristics

A total of 68 parents were invited to participate in this study. Among these parents, three disagreed to take part in this survey. Characteristics of the included 65 parents and their children are displayed in Table 1 and Figure 1. Figure S1 demonstrates the proportion of children in each age group who missed school or early childhood education between March 2020 and March 2022.

**TABLE 1:** Sociodemographic and clinical characteristics of parents and children.

Characteristics of parents	Values
Number of respondents [*n*]	65
Median age (Q25/Q75; min/max) [years]	38 (34/42; 23/56)
Gender of respondents (male/female) [*n* (%)]	12 (18%)/53 (82%)
Caregiver household composition	
Two caregivers, cohabiting [*n* (%)]	46 (71%)
Two caregivers, separated [*n* (%)]	12 (18%)
Single caregiver [*n* (%)]	7 (11%)
Household with more than one child [*n* (%)]	52 (80%)
Educational qualification of respondents	
Lower secondary or less [*n* (%)]	10 (15%)
Upper secondary [*n* (%)]	14 (22%)
Vocational training [*n* (%)]	29 (45%)
University degree [*n* (%)]	12 (18%)
Employment status of respondents	
Employed [*n* (%)]	48 (74%)
Not employed [*n* (%)]	17 (26%)

Characteristics of children	Values
Number of children [*n*]	65
Median age (Q25/Q75; min/max) [years]	6 (4/11.5; 4/18)
Gender of children (male/female) [*n* (%)]	33 (52%)/32 (48%)
Therapies and treatments of child	
Long‐term medication, any [*n* (%)]	42 (65%)
Antiseizure medication [*n*/42 (%)]	34 (81%)
Gastrointestinal/nutritional medication [*n*/42 (%)]	10 (24%)
Vitamins/minerals/supplements [*n*/42 (%)]	8 (19%)
Respiratory medication/oxygen therapy [*n*/42 (%)]	4 (10%)
Sleep‐regulating medication [*n*/42 (%)]	3 (7%)
Endocrine therapy [*n*/42 (%)]	2 (5%)
Muscle tone/spasticity medication [*n*/42 (%)]	1 (2%)
Speech therapy [*n* (%)]	51 (78%)
Physiotherapy [*n* (%)]	44 (68%)
Occupational therapy [*n* (%)]	42 (65%)
Type of educational institution of child	
Kindergarten [*n* (%)]	36 (55%)
School [*n* (%)]	3 (5%)
Special needs school [*n* (%)]	26 (40%)
Period of absence from the educational institution due to closure during the pandemic according to the parents' reports	
No absence [*n* (%)]	14 (22%)
1–7 weeks of absence [*n* (%)]	16 (25%)
8 or more weeks of absence [*n* (%)]	35 (53%)

*Note:* Multiple categories were possible within ‘Therapies and treatments of child’.

Abbreviations: min/max, minimum/maximum; Q25/Q75, quantile 25/quantile 75.

**FIGURE 1:**
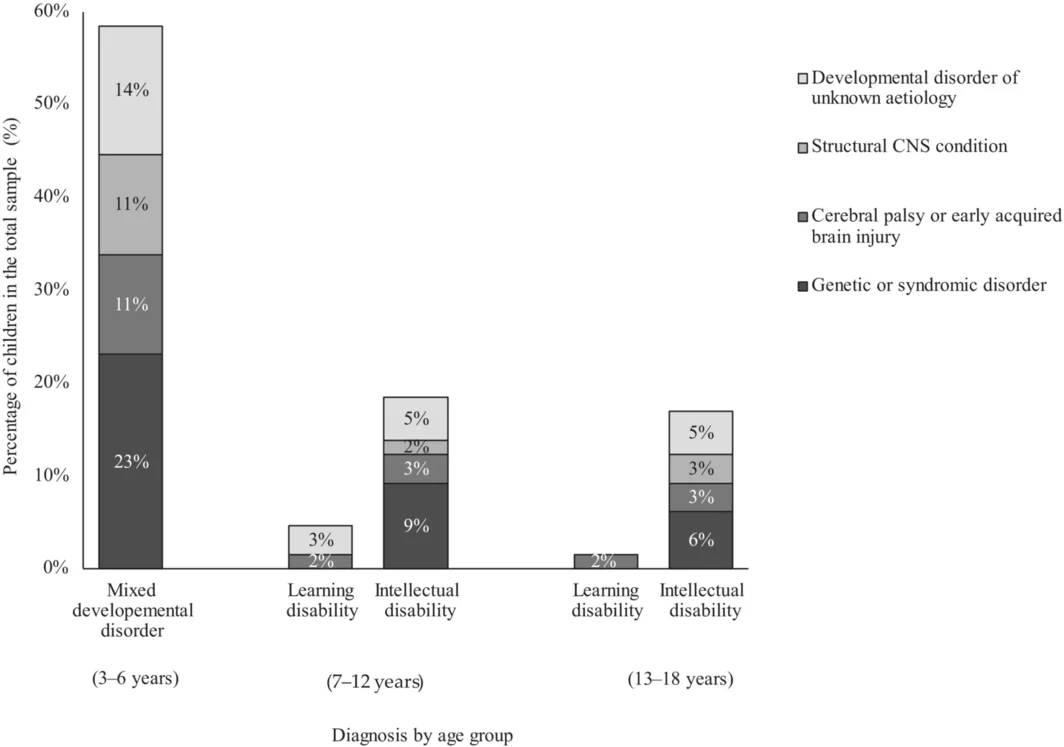
Distribution of developmental diagnoses and underlying conditions by age group in the total sample (*n* = 65).

### 3.2 Perceived Impact of the COVID‐19 Pandemic on the Families' and Children's Daily Life

Of the parents, 47/65 (72%) reported changes in their child's behaviour, development and/or health that they attributed to the pandemic. The remaining 18/65 (28%) parents noticed no or almost no changes in their children. Most frequently (multiple answers possible), parents mentioned negative emotional and behavioural changes (29/65; 45%). Details are shown in Table 2.

**TABLE 2:** Parental reports of pandemic‐related consequences for their child (multiple responses possible).

Consequences	Examples for quotes by parents	*n*/65 (%)
Emotional and behavioural changes	‘She was not able to let off steam on the playground, she was very unsettled at home’. (child: 7 y, ID; respondent: F, SC, EMP); ‘To keep a distance from other children was the worst for her’. (child: 5 y, MDD; respondent: F, CC, EMP)	29 (45%)
Developmental and educational setbacks	‘She had no therapies, no support measures, no doctor appointments, and she would have needed them to make progress’. (child: 5 y, MDD; respondent: F, CC, EMP); ‘I believe he regressed during the time without school’. (child: 6 y, MDD; respondent: F, SC, UNEMP); ‘[…] during those years he should have made progress in language development, which was not possible at home’. (child: 3 y, MDD; respondent: F, CC, UNEMP)	17 (26%)
Social difficulties and problems in communication	‘The entire social development suffered from it. He was already quite distant, but the pandemic worsened it’. (child: 6 y, MDD; respondent: F, CC, UNEMP); ‘She could not read faces (because of the mask), and that made communication with other people difficult’. (child: 10 y, ID; respondent: F, CC, UNEMP)	9 (14%)
Other consequences	‘He gained weight because he was less active’. (child: 6 y, MDD; respondent: F, CC, EMP); ‘The transition back to normal life afterward was tough for him’. (child: 5 y, MDD; respondent: F, CC, EMP)	4 (6%)
Positive effects	‘He was ill less often because everyone wore masks and was more cautious’. (child: 4 y, MDD; respondent: F, CC, EMP); ‘Many things improved, for example, her physical development, because she was not as exhausted as usual’. (child: 4 y, MDD; respondent: M, CC, EMP)	3 (5%)
No or minimal consequences	‘It had no impact on my child because she did not even notice it’. (child: 3 y, MDD; respondent: F, CC, EMP)	18 (28%)

*Note:* Percentages do not sum to 100% due to multiple responses.

Abbreviations: CC, caregivers cohabiting in the household; EMP, employed; F, female; ID, intellectual disability; LD, learning disability; M, male; MDD, mixed developmental disorder; SC, single‐caregiver household; UNEMP, unemployed; y, years.

Parents rated the burden of the COVID‐19 pandemic on parent–child interaction and the adequacy of governmental support during that time using a 5‐point Likert scale. The most frequent response regarding the burden was *partly burdensome* (22/65; 34%), whereas governmental support was most often rated as *not adequate at all* (23/65; 35%). Parents' responses regarding the burden on parent–child interactions and the perceived adequacy of governmental support are illustrated in Figure 2.

**FIGURE 2:**
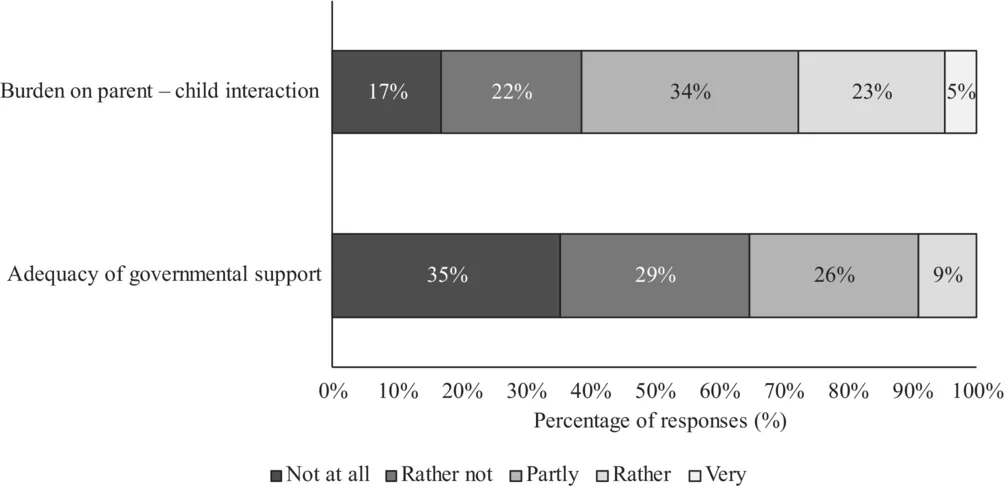
Parents' responses (*n* = 65) regarding perceived burden on parent–child interactions and the adequacy of governmental support during the COVID‐19 pandemic.

### 3.3 Perspectives on the Vaccination Against COVID‐19

Of the 65 children, 44/65 (68%) were eligible for vaccination (5 years or older, no COVID‐19 infection within the last 12 months). Among these children, parents of 18/44 (41%) decided in favour of vaccination, whereas parents of 26/44 (59%) decided against it. An overview of parental decisions regarding child COVID‐19 vaccination and reported reasons, benefits and disadvantages is shown in Figure 3.

**FIGURE 3:**
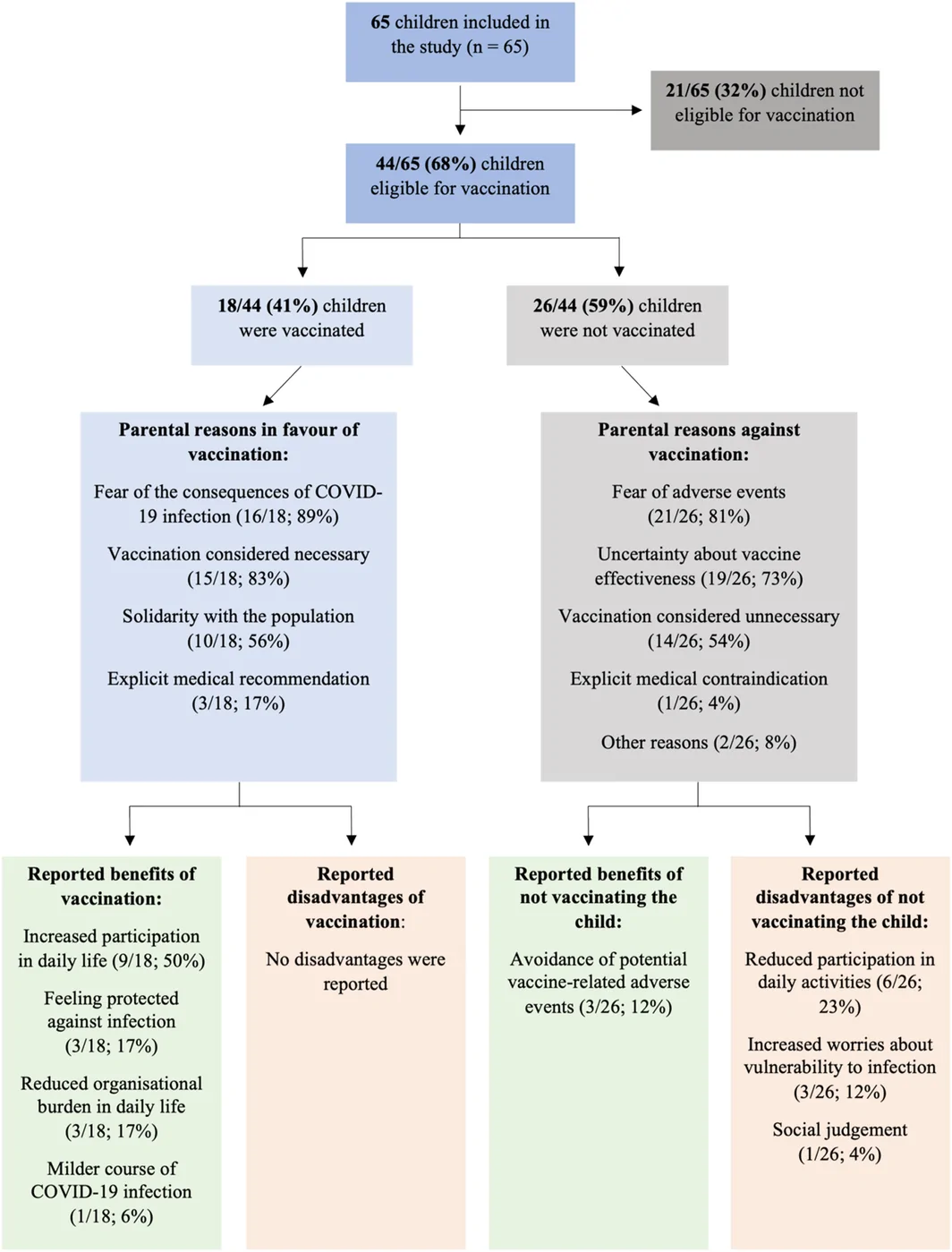
Child vaccination status, parental reasons in favour of or against vaccination and reported benefits and disadvantages of child vaccination among 65 parents. The flowchart summarises the vaccination status of the 65 participating children. Of these, 44 children were eligible for COVID‐19 vaccination, whereas 21 children were not eligible for vaccination at the time of the survey. Among eligible children, 18/44 (41%) had been vaccinated and 26/44 (59%) had not. The blue and grey boxes display the reported parental reasons for deciding in favour of or against vaccination. The green boxes present the perceived benefits of the respective decision, whereas the orange boxes summarise reported disadvantages. Percentages refer to the proportion of parents within the corresponding subgroup (vaccinated or nonvaccinated). Multiple answers were possible; therefore, percentages do not sum to 100%.

## 4 Discussion

The aim of this exploratory study was to gain insight into the experiences of families of children with developmental disorders of Northeastern Germany regarding the impact of the COVID‐19 pandemic on their daily lives. In addition, parents' attitudes towards COVID‐19 vaccination, as well as the consequences of these decisions, were examined.

Most parents reported negative consequences such as emotional and behavioural changes, developmental or educational setbacks or social difficulties and problems in communication. In retrospect, they attributed these changes to the pandemic. This finding is plausible, as daily life was fundamentally altered by school and kindergarten closures. Other measures, such as physical‐distancing rules, mask requirements and testing or quarantine protocols, also changed frequently (Onyeaka et al. 2021; Vygen‐Bonnet et al. 2022). These unpredictable changes demanded constant adaptation. However, stable structures and routines are crucial for optimal child development (Selman and Dilworth‐Bart 2023), and children with developmental disorders depend on them to an even greater extent (Summers et al. 2021). Moreover, limited cognitive flexibility and/or young age may have made it difficult for them to understand the sudden restrictions. Pre‐existing difficulties in emotion regulation and anxiety may have also increased their vulnerability to these disruptions (Reynolds et al. 2019; Spruyt and Curfs 2015). In addition, pandemic‐related routine changes have also been associated with increased screen time and reduced adherence to daily routines (Micheletti et al. 2021). Taken together, this may help contextualise the negative emotional and behavioural changes frequently described by parents in the present study. However, a causal association cannot be established based on this exploratory study. It remains unclear whether these consequences were specific to children with developmental disorders or reflected broader pandemic‐related challenges.

Another notable finding was that many parents reported developmental and educational setbacks in areas such as language, motor, cognitive and social development. Parents mainly linked these perceived consequences to the suspension of therapy sessions or reduced school or early childhood education attendance during the pandemic. During this period, children have had fewer opportunities to interact with peers and practise developmental skills. Similar challenges in remote education and reduced educational support for children with special educational needs and disabilities during the pandemic have been described from the perspective of teachers (Tzivinikou et al. 2021). Importantly, these effects may extend beyond the period of the pandemic. Accordingly, recent studies suggest that children may continue to experience developmental and school‐related challenges after the acute pandemic period, especially children with special educational needs and/or developmental disorders (Arbeau et al. 2025; Department for Education, n.d.; Totsika et al. 2024).

However, not all experiences were negative. About a quarter of parents with children with developmental disorders reported little or no consequences. A few even noticed positive changes, such as fewer infections, less exhaustion or perceived improvements in physical development, which they attributed to the pandemic. This aligns with other studies reporting similar experiences in families of children with intellectual disabilities and developmental disorders (Department for Education, n.d.; Totsika et al. 2024). Some children may have benefited from reduced pressure to perform at school, quieter daily routine, more self‐determined time at home or more time with their parents (Yusuf et al. 2022; Neece et al. 2020). However, other factors such as the child's maturation during the pandemic may also have contributed to the perceived positive changes, given the assumed association between age and behavioural and cognitive development (Mezzacappa et al. 1999).

Yet, the pandemic also placed a significant burden on parents. There was a rise in mental health issues, especially among those raising children with developmental disorders (Marchetti et al. 2020; Geweniger et al. 2022). This aligns with the present findings, as around 75% of the parents reported strained parent–child interactions. Furthermore, 64% of the parents judged the governmental support as either rather not adequate or not adequate at all. This may suggest that existing support measures in Germany, such as financial aids, hotlines or information campaigns, may not have been adequately tailored to the special needs of these families. Accordingly, another study conducted in Germany highlighted the unmet support needs and the importance of social support and social networks for parents of children with special healthcare needs during the COVID‐19 pandemic (Geweniger et al. 2024). However, without a control group, the present study cannot determine whether the extent of the burden differed from that experienced by families of typically developing children during the pandemic.

The availability of COVID‐19 vaccines offered hope for ending the pandemic, but also triggered debate (Kafadar et al. 2023; Kraaijeveld et al. 2022). Vaccine hesitancy is generally shaped by contextual, sociodemographic and individual factors, including cultural and socio‐economic background as well as perceptions of vaccination risks (Lazarus et al. 2024; Khatrawi and Sayed 2023). Parental decisions regarding paediatric COVID‐19 vaccination should therefore be interpreted within the recommendation context in the Year 2022. At that time, recommendations for children had only recently been introduced and were still changing, whereas evidence on vaccination in younger age groups was still developing. However, the recommendations for children with pre‐existing conditions such as developmental disorders did not change during the study period (Vygen‐Bonnet et al. 2022; Walter et al. 2022; Fowlkes et al. 2022; Watanabe et al. 2023). Parents of children with developmental disorders had to consider different aspects regarding the decision of having their child vaccinated or not (Tinker et al. 2021). On the one hand, these children often have underlying health conditions or a compromised immune system, which may place them at higher risk of getting infected or of severe illness if they were infected with COVID‐19 (Courtenay and Perera 2020; Landes et al. 2020; Tinker et al. 2021). Therefore, vaccination could have potentially offered them greater protection against the virus and its complications. This is reflected in the fact that the most frequently mentioned reason in favour of vaccination was the fear of the consequences of a COVID‐19 infection. Additionally, being vaccinated allowed the resumption of routine medical care, therapy sessions, social activities and school attendance. All these aspects are all essential for children's overall development and quality of life and are especially critical for children whose social participation and development is already impaired or endangered. Conversely, some parents considered the very same underlying condition and fragile immune system as reasons against vaccination, worrying about potential adverse events. Similar concerns have been reported both in the general population and among parents of children with neurodevelopmental conditions (Landes et al. 2020; Blackwell et al. 2025; Geweniger et al. 2024; Khatrawi and Sayed 2023; Walter et al. 2022). However, studies show lower COVID‐19 vaccine acceptance among parents of children with developmental disorders than among parents of neurotypical children (Al Saad et al. 2023; Khoodoruth et al. 2023). Research also shows that online misinformation (e.g., false links between COVID‐19 vaccines and autism) fuels distrust and concerns (Khorasani et al. 2023). Moreover, reported organisational challenges during the COVID‐19 vaccination rollout in Germany and other European countries, such as limited capacity and difficulties in appointment scheduling (European Centre for Disease Prevention and Control (ECDC) 2021), may have posed additional obstacles to vaccination. In addition, despite their elevated risk, individuals with developmental disorders were not consistently prioritised as a target group (Redquest et al. 2023; Baksh et al. 2021). This factor may also be relevant when interpreting lower vaccination uptake in this population. Therefore, future research is needed to develop vaccination campaigns for this population, which should involve trusted physicians, address specific parental concerns and counter misinformation in the community.

Limitations: Generalisability of this exploratory study may be limited due to being conducted at a single university hospital in Northeastern Germany. In addition, recruitment through a social paediatric centre may have introduced selection bias, as children treated in this setting often have more complex needs. Due to the small and heterogeneous sample, possible differences between mixed developmental disorders, learning disability and intellectual disability could not be examined in detail. Recall bias and subjective perception must be taken into account, as all main findings are based on parental reports and retrospective attribution. No standardised developmental or psychological assessments were used, and the questionnaire was developed specifically for this exploratory study and was not formally validated. Pilot testing was conducted with two parents of typically developing children and not with parents from the target population. No control group of parents of typically developing children was included, because the study aimed to provide descriptive insights into families of children with developmental disorders rather than to compare groups. Therefore, the findings cannot determine whether the reported experiences were specific to this population or reflected broader pandemic‐related challenges. Future studies should include control groups to distinguish between general family challenges and those specific to families of children with developmental disorders. Moreover, the interviews were conducted in the Year 2022, and the findings must be interpreted in light of the local policy context and the changing vaccination recommendations that applied at that time. The findings should also be interpreted considering the age range of the present sample, as evidence on pandemic‐related experiences among older adolescents and adults with developmental disorders remains limited.

## 5 Conclusions

In Northeastern Germany, many parents of children with developmental disorders perceived negative changes in their child's development, behaviour and emotional well‐being during the COVID‐19 pandemic and attributed these changes to the pandemic. Parental burden was perceived as high, whereas governmental support was frequently rated as not adequate. Among children eligible for COVID‐19 vaccination, parental decisions were mixed, and concerns about adverse events were common at a time when recommendations for paediatric vaccination were still evolving. Future research is needed to develop tailored vaccination campaigns and safety‐focused communication strategies for families of children with developmental disorders.

## Author Contributions

**Teresa Vela Martín:** conceptualization, methodology, formal analysis, investigation, writing – original draft. **Martina P. Neininger:** methodology, formal analysis, writing – review and editing. **Gudrun E. Krause:** methodology, supervision, writing – review and editing. **Thilo Bertsche:** methodology, writing – review and editing. **Astrid Bertsche:** funding acquisition, conceptualization, methodology, formal analysis, supervision, writing – review and editing, project administration. **Sarah Jeschke:** conceptualization, methodology, formal analysis, supervision, project administration, writing – original draft.

## Funding

The research was funded in part by the Federal Ministry of Education and Research (Bundesministerium für Bildung und Forschung, BMBF) as part of the German Center for Child and Adolescent Health (DZKJ) under the funding code 01GL2403A.

## Ethics Statement

The study was conducted in accordance with the Declaration of Helsinki and approved by the Ethics Committee of the University of Rostock (Registration Number: A 2022‐0067, April 2022).

## Consent

Written informed consent was obtained from parents.

## Conflicts of Interest

The authors declare no conflicts of interest.

## Supporting information


**Figure S1:** Proportion of children in each age group who missed school or early childhood education between March 2020 and March 2022.

## Data Availability

The data presented in this study are available on request from the corresponding author. The data are not publicly available due to ethical and privacy considerations to protect the confidentiality of participants.

## References

[cch70370-bib-0001] Aishworiya, R. , and Y. Q. Kang . 2021. “Including Children With Developmental Disabilities in the Equation During the COVID‐19 Pandemic.” Journal of Autism and Developmental Disorders 51: 2155–2158. 10.1007/s10803-020-04670-6. PMC7438977 32816170

[cch70370-bib-0002] Al Saad, A. J. , G. M. Alhassan , M. S. Albedaiwi , F. F. Alqattan , F. A. Aleisa , and H. W. Alabdulmuhsin . 2023. “Acceptance of COVID‐19 Vaccination Among Parents of Children With Autism and Other Neurodevelopmental Disorders in Saudi Arabia: A Cross‐Sectional Study.” BMC Public Health 23: 1235. 10.1186/s12889-023-16127-3. PMC10294403 37365523

[cch70370-bib-0003] Arbeau, K. , S. Atallah , and J. St. Pierre . 2025. “Families of Children/Youth With Complex Needs Before, During, and After COVID‐19 Pandemic Restrictions.” Journal of Child & Adolescent Trauma 18: 497–514. 10.1007/s40653-024-00676-9. PMC12130422 40469790

[cch70370-bib-0004] Baksh, R. A. , S. E. Pape , J. Smith , and A. Strydom . 2021. “Understanding Inequalities in COVID‐19 Outcomes Following Hospital Admission for People With Intellectual Disability Compared to the General Population: A Matched Cohort Study in the UK.” BMJ Open 11: e052482. 10.1136/bmjopen-2021-052482. PMC8491000 34607870

[cch70370-bib-0005] Blackwell, C. K. , T. Bekelman , S. Bakre , et al. 2025. “Sociodemographic Differences in Parental Hesitancy to the COVID‐19 Vaccine.” Vaccine 55: 127041. 10.1016/j.vaccine.2025.127041. PMC12078000 40132320

[cch70370-bib-0006] Bonuck, K. , S. Iadarola , Q. Gao , and J. F. Siegel . 2022. “COVID‐19 Vaccines for Children With Developmental Disabilities: Survey of New York State Parents' Willingness and Concerns.” Journal of Developmental and Behavioral Pediatrics 43: 521–528. 10.1097/DBP.0000000000001113. PMC9698114 36067424

[cch70370-bib-0007] Brandstetter, S. , M. Pawellek , M. M. Böhmer , et al. 2022. “Parental Intention to Vaccinate Their Children Against COVID‐19.” Bundesgesundheitsblatt, Gesundheitsforschung, Gesundheitsschutz 65: 1281–1288. 10.1007/s00103-022-03613-z. PMC9645347 36350345

[cch70370-bib-0008] Bussières, E. L. , C. Malboeuf‐Hurtubise , A. Meilleur , et al. 2021. “Consequences of the COVID‐19 Pandemic on Children's Mental Health: A Meta‐Analysis.” Frontiers in Psychiatry 12: 691659. 10.3389/fpsyt.2021.691659. PMC8672800 34925080

[cch70370-bib-0009] Courtenay, K. , and B. Perera . 2020. “COVID‐19 and People With Intellectual Disability: Impacts of a Pandemic.” Irish Journal of Psychological Medicine 37: 231–236. 10.1017/ipm.2020.45. PMC7287305 32404232

[cch70370-bib-0010] Department for Education . n.d. “Pupil Absence in Schools in England: 2024 to 2025.” Accessed on 30 June 2026. https://explore‐education‐statistics.service.gov.uk/find‐statistics/pupil‐absence‐in‐schools‐in‐england/2024‐25.

[cch70370-bib-0011] European Centre for Disease Prevention and Control (ECDC) . 2021. “Rollout of COVID‐19 Vaccines in the EU/EEA: Challenges and Good Practice; Public Health Guidance.” ECDC: Stockholm, Sweden. Accessed on 11 January 2026. https://www.ecdc.europa.eu/en/publications‐data/rollout‐covid‐19‐vaccines‐eueea‐challenges‐and‐good‐practice.

[cch70370-bib-0012] Fowlkes, A. L. , S. K. Yoon , K. Lutrick , et al. 2022. “Effectiveness of 2‐Dose BNT162b2 (Pfizer BioNTech) mRNA Vaccine in Preventing SARS‐CoV‐2 Infection Among Children Aged 5–11 Years and Adolescents Aged 12–15 Years—PROTECT Cohort, July 2021–February 2022.” MMWR. Morbidity and Mortality Weekly Report 71: 422–428. 10.15585/mmwr.mm7111e1. PMC8942308 35298453

[cch70370-bib-0013] Galanis, P. , I. Vraka , O. Siskou , O. Konstantakopoulou , A. Katsiroumpa , and D. Kaitelidou . 2022. “Willingness, Refusal and Influential Factors of Parents to Vaccinate Their Children Against the COVID‐19: A Systematic Review and Meta‐Analysis.” Preventive Medicine 157: 106994. 10.1016/j.ypmed.2022.106994. PMC8861629 35183597

[cch70370-bib-0014] Gardiner, E. , A. R. Miller , and L. M. Lach . 2018. “Family Impact of Childhood Neurodevelopmental Disability: Considering Adaptive and Maladaptive Behaviour.” Journal of Intellectual Disability Research 62: 888–899. 10.1111/jir.12547. 30230656

[cch70370-bib-0015] Geweniger, A. , M. Barth , A. D. Haddad , et al. 2022. “Impact of the COVID‐19 Pandemic on Mental Health Outcomes of Healthy Children, Children With Special Health Care Needs and Their Caregivers—Results of a Cross‐Sectional Study.” Frontiers in Pediatrics 10: 759066. 10.3389/fped.2022.759066. PMC8866820 35223688

[cch70370-bib-0016] Geweniger, A. , M. Barth , A. D. Haddad , et al. 2024. “Perceived Social Support and Characteristics of Social Networks of Families With Children With Special Healthcare Needs Following the COVID‐19 Pandemic.” Frontiers in Public Health 12: 1322185. 10.3389/fpubh.2024.1322185. PMC10937572 38487183

[cch70370-bib-0017] Grote, U. , M. Arvand , S. Brinkwirth , et al. 2021. “Maßnahmen zur Bewältigung der COVID‐19‐Pandemie in Deutschland: Nichtpharmakologische und Pharmakologische Ansätze [Measures to Cope With the COVID‐19 Pandemic in Germany: Nonpharmaceutical and Pharmaceutical Interventions].” Bundesgesundheitsblatt—Gesundheitsforschung—Gesundheitsschutz 64: 435–445. 10.1007/s00103-021-03306-z. PMC8010780 33787944

[cch70370-bib-0018] Heinsohn, T. , B. Lange , P. Vanella , et al. 2022. “Infection and Transmission Risks of COVID‐19 in Schools and Their Contribution to Population Infections in Germany: A Retrospective Observational Study Using Nationwide and Regional Health and Education Agency Notification Data.” PLoS Medicine 19, no. 12: e1003913. 10.1371/journal.pmed.1003913. PMC9767368 36538517

[cch70370-bib-0019] Houtrow, A. J. , C. S. Shearer , G. McKernan , K. Kuhlthau , and R. Agrawal . 2025. “Health Care Cost Concerns and Hardships for Families of Children With Disabilities.” JAMA Network Open 8: e257826. 10.1001/jamanetworkopen.2025.7826. PMC12022804 40272802

[cch70370-bib-0020] Irazábal, M. , F. Marsà , M. García , et al. 2012. “Family Burden Related to Clinical and Functional Variables of People With Intellectual Disability With and Without a Mental Disorder.” Research in Developmental Disabilities 33: 796–803. 10.1016/j.ridd.2011.12.002. 22245729

[cch70370-bib-0021] Kafadar, A. H. , G. G. Tekeli , K. A. Jones , B. Stephan , and T. Dening . 2023. “Determinants for COVID‐19 Vaccine Hesitancy in the General Population: A Systematic Review of Reviews.” Journal of Public Health (Berlin) 31: 1829–1845. 10.1007/s10389-022-01753-9. PMC9483252 36160668

[cch70370-bib-0022] Khatrawi, E. M. , and A. A. Sayed . 2023. “The Reasons Behind COVID‐19 Vaccination Hesitancy Among the Parents of Children Aged Between 5 to 11 Years Old in Saudi Arabia.” International Journal of Environmental Research and Public Health 20: 1345. 10.3390/ijerph20021345. PMC9859117 36674101

[cch70370-bib-0023] Khoodoruth, M. L. , L. O. Sabur , C. Melber , et al. 2023. “Evaluating COVID‐19 Vaccination Intentions and Vaccine Hesitancy Among Parents of Children With Autism Spectrum Disorder.” Scientific Reports 13: 7353. 10.1038/s41598-023-34191-y. PMC10161974 37147338

[cch70370-bib-0024] Khorasani, S. , A. S. Nacht , K. C. Goffin , et al. 2023. “A Qualitative Investigation on COVID‐19 Vaccine Hesitancy in Neurodivergent Communities.” Vaccine 11: 895. 10.3390/vaccines11050895. PMC10221563 37242999

[cch70370-bib-0025] Kraaijeveld, S. R. , R. Gur‐Arie , and E. Jamrozik . 2022. “Against COVID‐19 Vaccination of Healthy Children.” Bioethics 36: 687–698. 10.1111/bioe.13015. PMC9111802 35332941

[cch70370-bib-0026] Landes, S. D. , M. A. Turk , M. K. Formica , K. E. McDonald , and J. D. Stevens . 2020. “COVID‐19 Outcomes Among People With Intellectual and Developmental Disability Living in Residential Group Homes in New York State.” Disability and Health Journal 13: 100969. 10.1016/j.dhjo.2020.100969. PMC7311922 32600948

[cch70370-bib-0027] Lazarus, J. V. , T. M. White , K. Wyka , et al. 2024. “Influence of COVID‐19 on Trust in Routine Immunization, Health Information Sources and Pandemic Preparedness in 23 Countries in 2023.” Nature Medicine 30: 1559–1563. 10.1038/s41591-024-02939-2. PMC11186753 38684861

[cch70370-bib-0028] Li, Q. , Y. Li , J. Zheng , et al. 2023. “Prevalence and Trends of Developmental Disabilities Among US Children and Adolescents Aged 3 to 17 Years, 2018–2021.” Scientific Reports 13: 17254. 10.1038/s41598-023-44472-1. PMC10570352 37828147

[cch70370-bib-0029] Marchetti, D. , L. Fontanesi , C. Mazza , S. Di Giandomenico , P. Roma , and M. C. Verrocchio . 2020. “Parenting‐Related Exhaustion During the Italian COVID‐19 Lockdown.” Journal of Pediatric Psychology 45: 1114–1123. 10.1093/jpepsy/jsaa093. PMC7665691 33068403

[cch70370-bib-0030] Mezzacappa, E. , D. Kindlon , and F. Earls . 1999. “Relations of Age to Cognitive and Motivational Elements of Impulse Control in Boys With and Without Externalizing Behavior Problems.” Journal of Abnormal Child Psychology 27: 473–483. 10.1023/A:1021936210844. 10821629

[cch70370-bib-0031] Miao, R. , C. Liu , J. Zhang , and H. Jin . 2023. “Impact of the COVID‐19 Pandemic on the Mental Health of Children and Adolescents: A Systematic Review and Meta‐Analysis of Longitudinal Studies.” Journal of Affective Disorders 340: 914–922. 10.1016/j.jad.2023.08.070. 37598714

[cch70370-bib-0032] Micheletti, M. B. , P. Cafiero , S. Nápoli , et al. 2021. “Sleep Problems in a Sample of Children With Developmental Disorders, Before and During the COVID‐19 Pandemic.” Archivos Argentinos de Pediatría 119: 296–303. 10.5546/aap.2021.eng.296. 34569736

[cch70370-bib-0033] Neece, C. , L. L. McIntyre , and R. Fenning . 2020. “Examining the Impact of COVID‐19 in Ethnically Diverse Families With Young Children With Intellectual and Developmental Disabilities.” Journal of Intellectual Disability Research 64: 739–749. 10.1111/jir.12769. PMC7461180 32808424

[cch70370-bib-0034] Onyeaka, H. , C. K. Anumudu , Z. T. Al‐Sharify , E. Egele‐Godswill , and P. Mbaegbu . 2021. “COVID‐19 Pandemic: A Review of the Global Lockdown and Its Far‐Reaching Effects.” Science Progress 104: 368504211019854. 10.1177/00368504211019854. PMC10454957 34061685

[cch70370-bib-0035] Purrmann, L. , L. J. Speichert , A. Bäuerle , et al. 2021. “COVID‐19 Vaccine for Children: Determinants and Beliefs Contributing to Vaccination Decision of Parents in Germany 2021/2022.” Vaccine 12: 20. 10.3390/vaccines12010020. PMC10820980 38250833

[cch70370-bib-0036] Redquest, B. , S. Andreasen , K. Turner , and C. A. McMorris . 2023. “The Impact of the COVID‐19 Pandemic on People With Neurodevelopmental Disabilities and Their Families.” In Developments in Neuroethics and Bioethics, edited by W. B. Gibbard , vol. 6, 221–246. Academic Press. 10.1016/bs.dnb.2023.05.007.

[cch70370-bib-0037] Reynolds, A. M. , G. N. Soke , K. R. Sabourin , et al. 2019. “Sleep Problems in 2‐ to 5‐Year‐Olds With Autism Spectrum Disorder and Other Developmental Delays.” Pediatrics 143: e20180492. 10.1542/peds.2018-0492. PMC6398427 30745433

[cch70370-bib-0038] Schieve, L. A. , V. Gonzalez , S. L. Boulet , et al. 2006. “Concurrent Medical Conditions and Health Care Use and Needs Among Children With Learning and Behavioral Developmental Disabilities, National Health Interview Survey, 2006‐2010.” Research in Developmental Disabilities 33: 467–476. 10.1016/j.ridd.2011.10.008. 22119694

[cch70370-bib-0039] Selman, S. B. , and J. E. Dilworth‐Bart . 2023. “Routines and Child Development: A Systematic Review.” Journal of Family Theory & Review 16: 272–328. 10.1111/jftr.12549.

[cch70370-bib-0040] Sombetzki, M. , P. Lücker , M. Ehmke , et al. 2021. “Impact of Changes in Infection Control Measures on the Dynamics of COVID‐19 Infections in Schools and Pre‐Schools.” Frontiers in Public Health 9: 780039. 10.3389/fpubh.2021.780039. PMC8720754 34988054

[cch70370-bib-0041] Spruyt, K. , and L. M. Curfs . 2015. “Non‐Pharmacological Management of Problematic Sleeping in Children With Developmental Disabilities.” Developmental Medicine and Child Neurology 57: 120–136. 10.1111/dmcn.12623. 25370592

[cch70370-bib-0042] Summers, J. , D. Baribeau , M. Mockford , et al. 2021. “Supporting Children With Neurodevelopmental Disorders During the COVID‐19 Pandemic.” Journal of the American Academy of Child and Adolescent Psychiatry 60: 2–6. 10.1016/j.jaac.2020.09.011. 32998024

[cch70370-bib-0043] Tinker, S. C. , M. E. Cogswell , G. Peacock , and A. B. Ryerson . 2021. “Important Considerations for COVID‐19 Vaccination of Children With Developmental Disabilities.” Pediatrics 148: e2021053190. 10.1542/peds.2021-053190. PMC8883287 34272340

[cch70370-bib-0044] Totsika, V. , A. Kouroupa , A. Timmerman , et al. 2024. “School Attendance Problems Among Children With Neurodevelopmental Conditions One Year Following the Start of the COVID‐19 Pandemic.” Journal of Autism and Developmental Disorders 54: 2998–3007. 10.1007/s10803-023-06025-3. PMC11300561 37480438

[cch70370-bib-0045] Turk, M. A. , S. D. Landes , M. K. Formica , and K. D. Goss . 2020. “Intellectual and Developmental Disability and COVID‐19 Case‐Fatality Trends: TriNetX Analysis.” Disability and Health Journal 13: 100942. 10.1016/j.dhjo.2020.100942. PMC7245650 32473875

[cch70370-bib-0046] Tzivinikou, S. , G. Charitaki , and D. Kagkara . 2021. “Distance Education Attitudes (DEAS) During Covid‐19 Crisis: Factor Structure, Reliability and Construct Validity of the Brief DEA Scale in Greek‐Speaking SEND Teachers.” Technology, Knowledge and Learning 26: 461–479. 10.1007/s10758-020-09483-1.

[cch70370-bib-0047] van de Berg, S. , L. Coyer , U. von Both , et al. 2024. “Coverage and Determinants of COVID‐19 Child Vaccination in Munich, Germany, October 2022–January 2023: Results of the COVIP‐Virenwächter Study.” European Journal of Pediatrics 183: 3727–3738. 10.1007/s00431-024-05617-0. 38850330

[cch70370-bib-0048] Vygen‐Bonnet, S. , J. Schlaberg , and J. Koch . 2022. “Rolle, Arbeitsweise und Empfehlungen der Ständigen Impfkommission (STIKO) im Kontext der COVID‐19‐Pandemie [Role, Working Methods and Recommendations of the Standing Committee on Vaccinations (STIKO) in the Context of the COVID‐19 Pandemic].” Bundesgesundheitsblatt, Gesundheitsforschung, Gesundheitsschutz 65, no. 12: 1251–1261. 10.1007/s00103-022-03610-2. PMC9684750 36416888

[cch70370-bib-0049] Walter, E. B. , K. R. Talaat , C. Sabharwal , et al. 2022. “Evaluation of the BNT162b2 Covid‐19 Vaccine in Children 5 to 11 Years of Age.” New England Journal of Medicine 386: 35–46. 10.1056/NEJMoa2116298. PMC8609605 34752019

[cch70370-bib-0050] Watanabe, A. , R. Kani , M. Iwagami , H. Takagi , J. Yasuhara , and T. Kuno . 2023. “Assessment of Efficacy and Safety of mRNA COVID‐19 Vaccines in Children Aged 5 to 11 Years: A Systematic Review and Meta‐Analysis.” JAMA Pediatrics 177: 384–394. 10.1001/jamapediatrics.2022.6243. PMC9871947 36689319

[cch70370-bib-0051] Yusuf, A. , N. Wright , M. Steiman , et al. 2022. “Factors Associated With Resilience Among Children and Youths With Disability During the COVID‐19 Pandemic.” PLoS ONE 17: e0271229. 10.1371/journal.pone.0271229. PMC9337662 35905110

