# Supplementary material for: The COVID‐19 Pandemic Experience of Families With Children With Developmental Disorders in Northeastern Germany
Source: Child Care Health Dev. 2026 Nov 3;52(6):e70370. doi: 10.1111/cch.70370 (PMC13729080; doi:10.1111/cch.70370)
Supplement: Supplementary file 1 [file CCH-52-e70370-s001.pdf]

Supporting Information

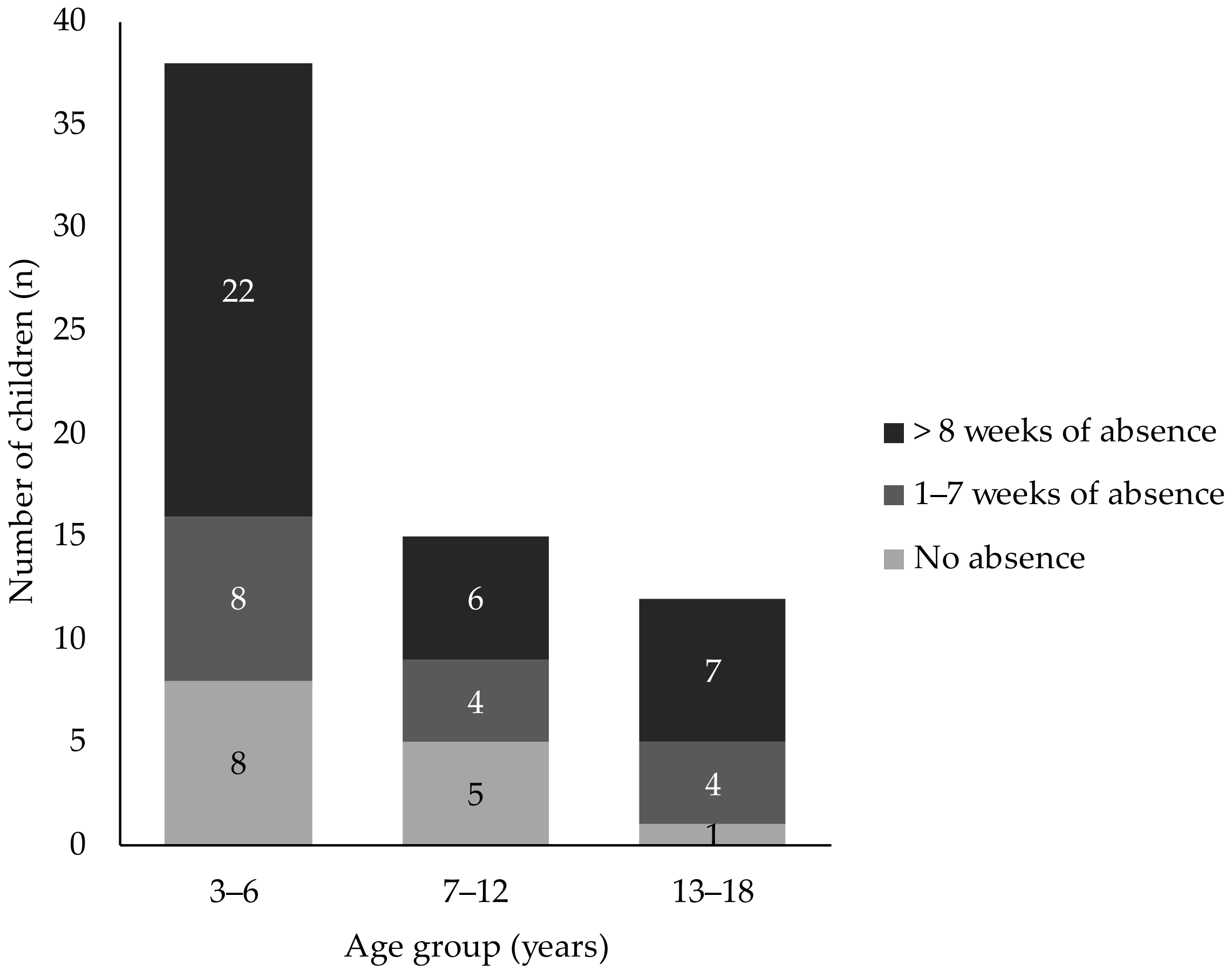

Figure S1. Proportion of children in each age group who missed school or early childhood education between March 2020 and March 2022.
